# White Blood Cells in Patients Treated with Programmed Cell Death-1 Inhibitors for Non-small Cell Lung Cancer

**DOI:** 10.1007/s00408-021-00474-2

**Published:** 2021-09-13

**Authors:** A. Sibille, M. Henket, J. L. Corhay, R. Alfieri, R. Louis, B. Duysinx

**Affiliations:** 1 Department of Pulmonology, University Hospital of Liège, Domaine de l’Université B35, 4000 Liège, Belgium; 2 Department of Pulmonology, Laboratory Unit of Pulmonology, University Hospital of Liège, Domaine de l’Université B35, 4000 Liège, Belgium; 3 Department of Internal Medicine, University Hospital of Liège, Domaine de l’Université B35, 4000 Liège, Belgium

**Keywords:** White blood cells, PD-1 inhibitors, Non-small cell lung cancer, Prognostic marker, Predictive marker, Checkpoint inhibitors

## Abstract

**Purpose:**

To investigate whether eosinophils and other white blood cell subtypes could be used as response and prognostic markers to anti-Programmed cell Death-1 or anti-PD-Ligand-1 treatments in non-small cell lung cancer patients.

**Methods:**

We retrospectively analyzed data from the NSCLC patients consecutively treated at our hospital with a PD-1/PD-L1 inhibitor in monotherapy for advanced disease. A total of 191 patients were evaluated at three time-points to investigate any relation between tumor response and WBC counts.

**Results:**

Baseline WBC and subtypes did not differ according to the type of response seen under treatment. A higher relative eosinophil count (REC) correlated with more objective responses (*p* = 0.019 at t1 and *p* = 0.014 at t2; OR for progression = 0.54 and 0.53, respectively) independently of the smoking status, PD-L1 status, and immune-related toxicity (IRT). Higher REC was also associated with a longer duration of treatment (*p* = 0.0096). Baseline absolute neutrophil count was prognostic (*p* = 0.049). At t1 relative lymphocytes, absolute and relative neutrophils, and neutrophil-to-lymphocyte ratio were prognostic (*p* = 0.044, *p* = 0.014, *p* = 0.0033, and *p* = 0.029, respectively).

**Conclusion:**

Our results show that in NSCLC patients anti-PD-1/PD-L1 therapy induces an early increase only in blood eosinophils, more prominent in responding patients and independent of the smoking status, PD-L1 status, and IRT. Eosinophils are also associated with a longer duration of treatment. Furthermore, our data support a prognostic role of neutrophils, lymphocytes, and their ratio for NSCLC patients with advanced disease treated with PD(L)-1 blockade.

## Introduction

The use of immune checkpoint inhibitors (ICI) for non-small-cell lung cancer (NSCLC) is increasing. Currently validated indications include advanced and locally advanced disease [1]. One of the challenges regarding ICI lies in the evaluation of objective response to these drugs. Classically, response evaluation relies on radiological criteria based on the Response Evaluation Criteria In Solid Tumors (RECIST) [2]. However, in the setting of ICI, these criteria seem imperfect. Indeed, several atypical response patterns like pseudoprogression have been observed that make radiological evaluation less clear than it is with chemotherapy [3]. In the search for additional evaluation tools, white blood cell (WBC) count has been investigated, among others, in melanoma and in NSCLC patients treated with Programmed cell Death (PD) Ligand (L)-1 inhibitors [4–7]. Some reports also mention a potential prognostic role of WBC subtypes and/or their ratio for various malignancies among which NSCLC [5, 7–9]. We previously reported a retrospective study investigating peripheral blood eosinophil counts as a parameter in the evaluation of response in NSCLC patients receiving PD-1 blockers [10]. In the present study we first aimed to investigate whether the results obtained in our former cohort could be confirmed. Then, we compared the potential predictive value of different subtypes of WBC and investigated the prognostic value of baseline WBC subtypes.

## Material and Methods

### Patients

All consecutive cases of advanced stage NSCLC were collected from our internal cancer registry from August 1st, 2015 to September 30th, 2019. A computer-based search was performed with the following inclusion criteria: (1) use of an anti-PD-1 or anti-PD-L1 agent (pembrolizumab at 2 mg/kg/3 weeks during the early access program (EAP) and then at 200 mg/3 weeks; nivolumab at 3 mg/kg/2 weeks during the EAP and then at 240 mg/2 weeks; atezolizumab at 1200 mg/3 weeks; durvalumab at 10 mg/kg/2 weeks) or (2) a pathological diagnosis of NSCLC for which the patient was registered in the electronic treatment prescription system. For the 388 patients identified the following exclusion criteria were applied: histology other than NSCLC (*n* = 27), missing laboratory values (*n* = 15), loss of follow-up (*n* = 22); early treatment discontinuation, i.e., before the second evaluation (*n* = 106) due to death, toxicity, and progressive disease without death or patient’s will; ongoing treatment (*n* = 7) or chemotherapy combined with anti-PD-1 (*n* = 20). Based on this, 191 patients were included in the present analysis.

### Data Collection

We collected the following data: (i) *patient characteristics*: age at the start of immunotherapy, gender, smoking status, concomitant obstructive airway disease, use of inhaled or oral corticoids and the reason for it (underlying respiratory condition, immune-related toxicity (IRT), other), date of death, and baseline Eastern Cooperative Oncology Group (ECOG)-Performance Status (PS); (ii) *lung cancer characteristics*: histology, stage of disease, line of treatment of the anti-PD (L)-1, PD-L1 expression level, based on immunohistochemistry (monoclonal antibody clone 22C3 with Automated Stainer, Dako), and presence or absence of a mutation based on next-generation sequencing analysis and ALK immunohistochemistry; (iii) *treatment characteristics*: dates of the start of treatment [t0], first evaluation (t1) and second evaluation (t2), immunotherapeutic agent, response at t1 and t2 using the RECIST criteria v1.1, immune-related toxicity (IRT), and duration of treatment; (iv) *biological variables*: total WBC counts and differential WBC counts (neutrophils, lymphocytes, eosinophils; absolute and relative) at t0, t1, and t2.

### Response Evaluation

A total of 191 patients were assessed for tumor response based on the RECIST criteria v 1.1 at two time-points (t1, t2; 8 to 12 weeks interval in between) and compared with baseline data. We describe patients as responders (R; for complete or partial response), stable (S), or progressive (P). We focused on the first two radiological evaluations as the majority of objective responses occur in the first two months of treatment (t1) with PD-1 blockers in monotherapy for NSCLC [11, 12]. We extended the evaluation period to the second radiological evaluation (t2) in order to include the patients showing a non-significant response at t1 further evolving toward progression or response.

### Duration of Treatment

Duration of treatment with anti-PD (L)-1 drugs was calculated from the time of first administration until the last recorded dose administration (data cut-off December 5th, 2019) and expressed in weeks.

### Overall Survival

Overall survival (OS) was defined as the time between the first dose of PD (L)-1 blocker and the date of death from any cause and expressed in months. If still alive at data cut-off (December 5th, 2019) the patient was censored.

### Statistical Analyses

Biological variables were studied as continuous variables and are described as medians and interquartile ranges. Qualitative data are described using frequencies and percentages. For the analyses on biological variables logarithmic analyses were performed (translated logarithm log (. +1) for the relative eosinophil count (REC) in percentage and log (. +0.01) for the absolute eosinophil count (AEC) in 10^3^cells/mm^3^). Univariate logistic regression analyses were performed with determination of the Odds ratio (OR), with confidence interval (CI) at 95% and p-values. Survival was calculated, expressed in months, and reported with Kaplan–Meier curves, and Cox regression models were used to analyze the impact of the different variables on the survival and reported as Hazard Ratio (HR), with CI at 95% and p-values. Results were considered significant with an uncertainty level of 5% (*p* < 0.05). Calculations were made with the help of SAS software (version 9.4) and graphs with R software (version 3.6.2).

## Results

### Patient Characteristics

A total of 191 patients were included in the study (Table 1). Approximately two-thirds of the patients were male with a large majority (94.8%) of (former) smokers and in good performance status (PS; 92.7% PS 0–1). Slightly more than half of the patients presented with a chronic obstructive airway disease at the time of PD(L)-1 blocker initiation but only 10.5% used inhaled corticoids and none used oral corticoids during the study period. The predominant histology was adenocarcinoma (55.5%). The majority of patients (69.7%) had stage IV disease at the time of treatment with PD(L)-1 blockade. Most of the patients (67.7%) received an anti-PD(L)-1 antibody in second or later line of treatment.

**Table 1:** Patient's characteristics

Characteristic	Total (*n* = 191)	Number (%)
Age-years		
Median		66
Range		42–85
Gender	191	
Male		122 (63.9)
Female		69 (36.1)
Smoking status	191	
Non-smoker		10 (5.2)
Former smoker		117 (61.3)
Current smoker		64 (33.5)
Obstructive airway disease	191	
None		83 (43.5)
COPD		88 (46.1)
Asthma		20 (10.4)
Inhaled corticosteroids	191	
No		171 (89.5)
Yes		20 (10.5)
ECOG-PS	191	
0		26 (13.6)
1		151 (79.1)
2+		14 (7.3)
Histology	191	
Adenocarcinoma		106 (55.5)
NOS		7 (3.7)
Squamous cell carcinoma		72 (37.7)
LCNE carcinoma		6 (3.1)
Oncogenic driver	119	
None		77 (64.7)
EGFR		3 (2.5)
ALK		0 (0)
Other		19 (16)
Unknown		20 (16.8)
Disease stage	191	
II		2 (1.0)
III		56 (29.3)
IV		133 (69.7)
PDL-1 category	191	
1		64 (33.5)
2		26 (13.6)
3		29 (15.2)
4		72 (37.7)
IT line stage IV	133	
1L		43 (32.3)
2L+		90 (67.3)
IT Agent	191	
Nivolumab		100 (52.3)
Pembrolizumab		58 (30.4)
Durvalumab		22 (11.5)
Atezolizumab		11 (5.8)

Smoking status: as registered at the start of PD(L)-1 blockade. Obstructive airway disease: *COPD* Chronic obstructive pulmonary disease, *ECOG-PS* Eastern Cooperative Oncology Group-Performance Status. Histology: *NOS* not otherwise specified. Oncogenic driver: *EGFR* Epidermal growth factor receptor (Tumor Hotspot Mastr kit, Illumina MiSeq), *ALK* Anaplastic lymphoma kinase (monoclonal antibody with Automated Stainer Benchmark, Roche), *other* BRAF, KRAS, and PIK3CA mutations (Tumor Hotspot Mastr kit, Illumina MiSeq), *unknown* No NGS or EGFR/ALK testing done, *no* At least no EGFR mutation/ALK rearrangement identified. Disease stage: according to the TNM 7th classification. PD-L1 category: *1* ≥ 50%, *2* 1–49%, *3* < 1%, *4* Unknown. IT line stage IV: line of treatment for the PD(L)-1 blockade: *1L* First line and *2L* + Second or later line

### White Blood Cell Counts Over Time Under PD(L)-1 Blockade

Baseline WBC and subtypes did not differ between responding, stable, and progressive patients. Among the studied biological variables only eosinophils rose under PD(L)-1 inhibition between the start of treatment and the time of first or second evaluation (*p* < 0.0001) (Table 2).

**Table 2:** Kinetics of white blood cell counts over time

	t0	t1	t2	*p*-value
WBC 10^3^cell/mm^3^	8.47 ± 3.70	8.09 ± 3.16	8.56 ± 4.94	0.70
Eosinophils %	2.34 ± 2.00^ab^	3.38 ± 2.79^a^	3.29 ± 2.83^b^	<0.0001
10^3^cell/mm^3^	0.19 ± 0.20^ab^	0.27 ± 0.27^a^	0.29 ± 0.40^b^	<0.0001
Lymphocytes %	20.16 ± 9.67	20.66 ± 8.54	20.41 ± 9.50	0.43
10^3^cell/mm^3^	1.56 ± 0.75	1.55 ± 0.62	1.58 ± 0.70	0.43
Neutrophils %	67.13 ± 11.93	65.68 ± 9.81	65.65 ± 13.32	0.20
10^3^cell/mm^3^	5.90 ± 3.28	5.47 ± 2.85	5.99 ± 4.72	0.15
NLR	4.64 ± 3.83	4.38 ± 5.26		0.20

*t0* Pre-treatment, *t1* First evaluation, *t2* Second evaluation. Comparisons made with Scheffé’s test between t0–t1 (*p*^a^ < 0.0001) and t0–t2 (*p*^b^ < 0.0001). WBC: white blood cells. *NLR* neutrophils-to-lymphocytes ratio

### Response

At the time of first evaluation 51 (26.7%) of the 191 patients were responders (R), 103 (53.9%) stable (S), and 37 (19.4%) progressive patients (P). At t2, we found 64 R (33.5%), 67 S (35.1%), and 60 P (31.4%). We found 3 patients (4.7%) showing progression at t1 but response at t2, so-called pseudoprogression. Five R (8.3%) became P at t2.

Higher response rates were noted for high PD-L1 expression (i.e., >50%; *p* = 0.0001 at t1 and *p* = 0.0031 at t2), pembrolizumab use (*p* < 0.0001 at t1 and *p* = 0.0096 at t2), and former smokers (*p* = 0.024; OR = 2.88).

Regarding biological variables none of the baseline values predicted the response at t1 or at t2. Responders had a significantly higher REC than progressive patients at t1 (*p* = 0.019 with OR = 0.54) and at t2 (*p* = 0.014 with OR = 0.53). By univariate analysis (two-way factorial ANOVA) PD-L1 status (*p* = 0.18 for REC and *p* = 0.067 for AEC), smoking status (*p* = 0.43 for REC and *p* = 0.13 for AEC) and immune-related toxicity (IRT) (*p* = 0.87 for REC and *p* = 0.93 for AEC) had no influence on eosinophil levels. No biological variable other than eosinophils was predictive of the response at t1 or at t2 (Table 3).

**Table 3:** Biological variables according to the type of response at t2

	Responders		Stable		Progressive	
*WBC*						
t0	8.53 (5.92–10.61)		7.76 (6.22–18.49)		7.68 (6.04–10.36)	
t1	6.82 (5.74–8.98)		7.79 (5.94–8.60)		7.66 (6.25–9.42)	
t2	6.63 (5.66–8.89)		7.58 (6.54–9.13)		8.52 (6.55–10.61)	
*Eosinophils*	*AEC*	*REC*	*AEC*	*REC*	*AEC*	*REC*
t0	0.14 (0.08–0.28)	1.85 (0.90–3.40)	0.13 (0.09–0.23)	1.90 (1.00–3.10)	0.12 (0.06–0.21)	1.65 (0.80–8.70)
t1	0.22 (0.14–0.35)	3.1 (2.05–4.75)*	0.2 (0.11–0.30)	2.9 (1.50–4.00)*	0.19 (0.10–0.34)	2.6 (1.30–3.60)
t2	0.24 (0.15–0.39)	3.55 (1.85–5.50)	0.22 (0.13–0.30)	2.50 (1.80–3.70)	0.13 (0.06–0.31)	1.90 (0.80–3.80)
*Neutrophils*	*ANC*	*RNC*	*ANC*	*RNC*	*ANC*	*RNC*
t0	5.51 (3.75–7.70)	68.75 (60.40–74.55)	5.06 (3.96–6.87)	66.60 (60.20–74.20)	5.31 (3.88–7.59)	68.95 (62.65–74.00)
t1	4.54 (3.67–5.77)	64.45 (56.75–69.70)	5.27 (3.76–5.98)	66.90 (62.00–72.10)	5.30 (4.03–6.63)	67.15 (60.85–74.65)
t2	4.14 (3.19–5.77)	63.90 (52.70–68.50)	5.07 (4.23–6.31)	67.60 (59.50–74.00)	5.95 (4.26–7.77)	69.35 (62.90–80.15)
*Lymphocytes*	*ALC*	*RLC*	*ALC*	*RLC*	*ALC*	*RLC*
t0	1.62 (1.13–2.02)	19.80 (14.25–25.15)	1.33 (1.04–1.85)	18.20 (13.60–24.40)	1.51 (1.09–1.89)	17.55 (14.40–25.10)
t1	1.55 (1.10–1.86)	21.25 (15.95–26.40)	1.45 (1.10–1.82)	19.90 (15.00–24.40)	1.48 (1.01–1.85)	19.95 (12.85–24.65)
t2	1.67 (1.17–2.06)	22.20 (16.75–29.30)	1.46 (1.15–1.84)	18.50 (13.80–24.90)	1.29 (1.03–1.89)	16.80 (10.75–24.95)

*WBC* White blood cells (.10^3^cell/mm^3^), *AEC* Absolute eosinophil count (.10^3^cell/mm^3^), *REC* Relative eosinophil count (%), *ANC* Absolute neutrophil count (.10^3^cell/mm^3^), *RNC* Relative neutrophil count (%), *ALC* Absolute lymphocyte count (.10^3^cell/mm^3^), *RLC* Relative lymphocyte count (%). Responders (*n* = 64), stable (*n* = 67), progressive (*n* = 60) patients: according to the RECIST criteria (see materials and methods). Results expressed as medians and interquartile ranges. Logistic regression analysis; *p*-value vs. progressive. ^*^p < .05

### Toxicity

The overall rate for IRT was 24.1%. Most IRT was of low intensity, requiring no immunosuppressive therapy. Indeed, only 12 out of 191 patients (6.3%) required oral corticoids (OCS) for the control of their IRT. Skin (22/191 patients, 11.6%), thyroid (9 patients, 4.7%), joints (5 patients, 2.6%), and lungs (5 patients, 2.6%) were the most frequently involved. For the durvalumab subgroup we identified significantly more IRT (40.9% reported at t1 or t2 vs. 21.9% for non-durvalumab drugs, *p* = 0.017), a higher use of OCS (18.1% vs. 4.7%, *p* = 0.0057), and higher pulmonary and thyroid toxicity (13.6% for both in the durvalumab group, compared to 1.2% and 3.6% for patients receiving non-durvalumab drugs, respectively; *p* = 0.0037). There was no correlation between baseline WBC subtypes and toxicity and no correlation between toxicity and response.

### Duration of Treatment

**Fig. 1:**
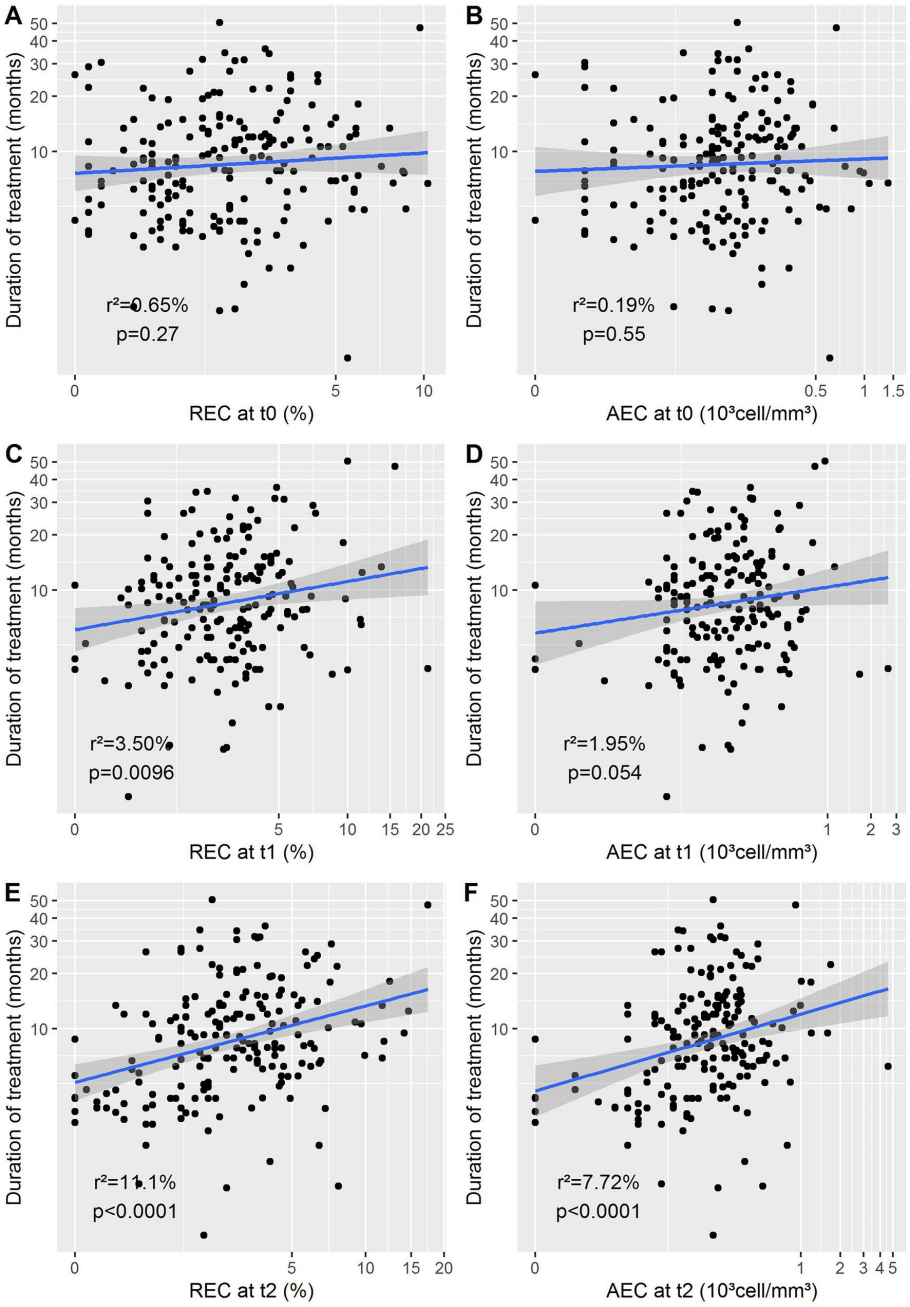
Eosinophils and duration of treatment. Logarithmic scale representation for relative eosinophil counts (REC) and absolute eosinophil counts (AEC). t0: before treatment; t1: at time of first evaluation; t2: at time of second evaluation

At the time of first evaluation a higher REC and a lower ANC were associated with a longer duration of treatment (*p* = 0.0096 and *p* = 0.021, respectively) (Fig. 1). At t2 all biological variables were predictive for the duration of treatment (data not shown).

### Overall Survival

The median OS was 18.8 months with 98 patients (51.3%) alive at data cut-off. No clinico-pathological feature was prognostic in this cohort. The OS was longer in patients responding at t1 (*p* < 0.0001) with medians of OS of 30.4 months for responders, 19.9 months for stable, and 12.8 months for progressive patients. A lower baseline absolute neutrophil count (ANC) correlated with longer OS (*p* = 0.049) while at t1, the relative lymphocyte count (RLC), relative neutrophil count (RNC), ANC, and neutrophil-to-lymphocyte ratio (NLR) were correlated with OS (*p* = 0.044, *p* = 0.014, *p* = 0.0033, and *p* = 0.029, respectively) (Table 4).

**Table 4:** Risk of death in the 191 patients according to biological variables at t1

Variable	t1 values	*p*-values	HR	95% CI
*White blood cells (10*^*3*^*cells/mm*^*3*^*)*				
Alive	7.28 (5.74–8.90)	0.094	1.78	0.91–3.51
Dead	7.80 (6.32–8.97)			
*Eosinophils (%)*				
Alive	2.90 (1.90–4.00)	0.081	0.70	0.47–1.04
Dead	2.80 (1.30–4.10)			
*Eosinophils (10*^*3*^*cells/mm*^*3*^*)*				
Alive	0.21 (0.12–0.33)	0.22	0.84	0.64–1.10
Dead	0.20 (0.11–0.33)			
*Lymphocytes (%)*				
Alive	20.85 (15.90–25.50)	0.044^*^	0.64	0.42–0.99
Dead	19.10 (13.80–23.00)			
*Lymphocytes (10*^*3*^*cells/mm*^*3*^*)*				
Alive	1.56 (1.10–1.88)	0.39	0.81	0.50–1.31
Dead	1.47 (1.09–1.81)			
*Neutrophils (%)*				
Alive	64.10 (59.40–69.20)	0.014^*^	1.03	1.00–1.05
Dead	68.90 (60.80–73.60			
*Neutrophils (10*^*3*^*cells/mm*^*3*^*)*				
Alive	4.62 (3.54–5.87)	0.0033^*^	1.11	1.04–1.20
Dead	5.21 (4.11–6.32)			
*NLR*				
Alive	2.96 (2.38–4.27)	0.029^*^	1.46	1.04–2.06
Dead	3.56 (2.67–5.35)			

Results expressed as medians and interquartile ranges. Alive (*n* = 98)/dead (*n* = 93): as recorded at data cut-off (see Materials and methods). *HR* Hazard ratio for death. *CI* Confidence interval. *NLR* Neutrophils to lymphocytes ratio. ^*^significant *p*-value (< 0.05)

## Discussion

In this cohort of advanced stage NSCLC patients treated with PD(L)-1 blockade, PD-L1 expression levels and smoking history were associated with response, confirming earlier data [13, 14]. Pembrolizumab was associated with more responses, as it was the only drug used in patients with high PD-L1 expression levels. Regarding biological data we noted an early rise only in eosinophils. Moreover, a higher proportion of eosinophils was associated with an early response and with a longer duration of treatment. Neutrophils, lymphocytes, and their ratio, either at baseline or early in the course of treatment, appeared to be prognostic.

The role of eosinophils in tumors is still a matter of debate. In various tumor types in vitro data and preclinical models show direct and indirect anti-tumor effects [15, 16] but also pro-tumorigenic effects [17–20]. Neutrophils can, like eosinophils, have both anti- and pro-tumor functions [21, 22].

The prognostic and predictive value of blood biomarkers and more specifically WBC and their subtypes in patients treated with ICI have been reported in several tumor types, e.g., colorectal cancer [23], breast cancer [24, 25], prostate cancer [26], melanoma [4, 27–29], and NSCLC [7]. However, these studies lack homogeneity: absolute vs. relative WBC counts, single vs. composite markers, and continuous vs. categorized variables. Weide and colleagues proposed a prognostic model based on categorized serum lactate dehydrogenase (LDH), WBC count, and clinical characteristics [4]. The risk of death was 2.4-fold (*p* = 0.003) and 2.2-fold (*p* < 0.001) for patients with pre-treatment RLC < 17.5% and REC < 1.5%, respectively. In part based on these results Tanizaki and colleagues studied the prognostic and predictive value of peripheral blood biomarkers in a population of NSCLC patients treated with nivolumab for advanced disease (*n* = 137) [7]. They found a strong association between baseline low (<7.5 cells/mL) ANC, high (>1.0 cells/mL) absolute lymphocyte count (ALC) and high (>0.15 cells/mL) AEC and higher response rates, progression-free survival (PFS), and OS. In those two studies, authors used categorized variables, i.e., AEC > or < 0.15 cells/mL and REC > or < 1.5%. We, however, considered the variables as continuous. Keeping this in mind, in our cohort a higher proportion in eosinophils at the time of the first evaluation (t1) were associated with a higher chance of objective response to treatment at t1 and t2. In our series, this was independent of the smoking history, PD-L1 status, and immune-related toxicity (IRT). We could, however, not identify a cut-off value for REC at t1 with satisfying sensitivity for discriminating responders from stable and from progressive patients at t2 (32.8% sensitivity and 81.9% specificity for a cut-off of 5.3% REC, *p*-value = 0.0137). Our study also emphasizes the association between blood eosinophils and the durability of clinical benefit for NSCLC patients, as expressed by the duration of treatment. A series of melanoma patients comfort these findings with response to ipilimumab correlated with an early rise in eosinophils [27].

In contrast to other series, toxicity in our cohort was not correlated with a higher probability of response and also not with raised eosinophils. Several retrospective studies [30–32] and one prospective report [33] showed an association between early IRT for advanced NSCLC and outcome. Although these studies are small sized and mostly lack pathological correlation, there is some rationale to explain this link: similarity between tumor antigens and self-antigens leading to cross-reactivity of T cells that are reactivated by the ICI [34], pre-existing autoimmunity with reactivation of T cells primarily directed at self-antigens [35] or B-cell reactivation through PD-1 blockade [36]. The fact that we did not find a correlation with response may be due to the retrospective nature of the study with incomplete data collection during patients’ follow-up. On the other hand, a correlation between eosinophilia (i.e., AEC > 0.5 cells/mL) and immune-related toxicity (*p* = 0.0042) has been demonstrated in a retrospective series including 146 patients with various solid tumor types treated with anti-PD(L)-1 [37]. As a correlation between eosinophils and response to ICI and between eosinophils and toxicity under ICI were shown, it is tempting to think that both clinical results (response and toxicity) are two sides of one phenomenon: immune (re-)activation. This, however, remains to be formally proven.

Some authors found a prognostic value of baseline eosinophils [4, 7]. This was not the case in our series. However, we found a clear association between eosinophils and response to treatment and between response and OS. The lack of prognostic value of REC at t0 may be due to small sample size when compared to the work of Weide and colleagues. Moreover, the prognostic value of baseline neutrophils, lymphocytes, and neutrophils/lymphocytes ratio (NLR) demonstrated in our work supports the findings of several authors [5, 8, 38]. Illustratively, the prognostic value of the iSEND model (immunotherapy, Sex, ECOG-PS, NLR, and Delta NLR) is being investigated in a prospective manner after it showed its value as a predictive tool for patients with advanced NSCLC treated with nivolumab [8). In earlier stages of disease a study on operated NSCLC specimen revealed an inverse correlation between neutrophils and CD8 + cytotoxic T cells [39].

An additional interesting finding of the present study is that blood eosinophils are the only WBC subtype displaying a rise during the first six months of anti-PD (L)-1 therapy for NSCLC, data that are in keeping with results from a large French cohort and from our previously published data [10, 40]. Further studies will have to explore why this rise is transient and whether raised eosinophils in responders are a consequence of or a trigger for immune anti-tumor activation.

## Conclusion

In this study patients receiving PD(L)-1 blockade for advanced NSCLC and showing a raised proportion of eosinophils at the time of first evaluation were more likely to show an objective response according to the RECIST criteria at the time of second evaluation, regardless of smoking history, PD-L1 status, and IRT. A higher REC also correlated with a longer duration of treatment. We could, however, not identify a clear cut-off value to propose eosinophils as a predictive biomarker. It seems necessary to identify the underlying mechanism(s) leading to a rise in blood eosinophils in patients deriving clinical benefit from anti-PD(L)-1 drugs. Further results of this cohort support the prognostic role of neutrophils, lymphocytes, and their ratio, either at baseline or early in the course of treatment.

## Data Availability

The datasets supporting the conclusions of this article are included within the article.
